# Advanced nanomaterials for imaging of eye diseases

**DOI:** 10.5599/admet.2182

**Published:** 2024-02-20

**Authors:** Van Phuc Nguyen, Justin Hu, Josh Zhe, Sanjay Ramasamy, Umayr Ahmed, Yannis M. Paulus

**Affiliations:** 1 Department of Ophthalmology and Visual Sciences, University of Michigan, Ann Arbor, MI 48105, USA; 2 Department of Biomedical Engineering, University of Michigan, Ann Arbor, MI 48105, USA

**Keywords:** Gold nanoparticles, contrast agents, nanomedicine, biosensors, ophthalmic imaging

## Abstract

**Background and purpose:**

Vision impairment and blindness present significant global challenges, with common causes including age-related macular degeneration, diabetes, retinitis pigmentosa, and glaucoma. Advanced imaging tools, such as optical coherence tomography, fundus photography, photoacoustic microscopy, and fluorescence imaging, play a crucial role in improving therapeutic interventions and diagnostic methods. Contrast agents are often employed with these tools to enhance image clarity and signal detection. This review aims to explore the commonly used contrast agents in ocular disease imaging.

**Experimental approach:**

The first section of the review delves into advanced ophthalmic imaging techniques, outlining their importance in addressing vision-related issues. The emphasis is on the efficacy of therapeutic interventions and diagnostic methods, establishing a foundation for the subsequent exploration of contrast agents.

**Key results:**

This review focuses on the role of contrast agents, with a specific emphasis on gold nanoparticles, particularly gold nanorods. The discussion highlights how these contrast agents optimize imaging in ocular disease diagnosis and monitoring, emphasizing their unique properties that enhance signal detection and imaging precision.

**Conclusion:**

The final section, we explores both organic and inorganic contrast agents and their applications in specific conditions such as choroidal neovascularization, retinal neovascularization, and stem cell tracking. The review concludes by addressing the limitations of current contrast agent usage and discussing potential future clinical applications. This comprehensive exploration contributes to advancing our understanding of contrast agents in ocular disease imaging and sets the stage for further research and development in the field.

## Introduction

Ocular diseases are a significant global concern, with an alarming projection of increasing vision impairment and blindness in the near future. It is estimated that by the year 2050, approximately 895 million individuals will be grappling with various degrees of vision impairment, out of which 61 million will be blind [1-3]. Causes include diseases such as age-related macular degeneration (AMD) [4,5], diabetic retinopathy [6], and retinitis pigmentosa [7]. To improve treatment outcomes, imaging-guided therapy is important to diagnose the disease at an early stage, leading to improved treatment planning and the potential to restore vision for patients [4]. This underscores the pressing need for innovative and advanced diagnostic techniques and treatment strategies in the field of ophthalmology.

In recent years, there has been a growing emphasis on the development of imaging modalities that can effectively diagnose and differentiate a myriad of ocular pathologies, ultimately facilitating early intervention and treatment [8-16]. The advent of advanced imaging technologies has ushered in a new era of precision in visualizing ocular structures and functions. These imaging techniques hold the promise of revolutionizing the way we understand, diagnose, and manage ocular diseases. Among the many imaging modalities in ophthalmology, optical coherence tomography (OCT) [17,18], OCT angiography (OCTA) [19,20], fluorescein angiography (FA) [21-23], and indocyanine green angiography (ICGA) [24,25] have all made significant clinical contributions to our ability to visualize the intricate vascular networks of the eye.

However, these modalities are not without their limitations, which have prompted researchers to explore novel avenues for enhancing the diagnostic utility of ocular imaging for future clinical applications. One such avenue lies in the integration of contrast agents. The application of contrast agents offers the possibility to augment the sensitivity and specificity of imaging techniques, enabling a deeper understanding of ocular pathologies at both the molecular and anatomical levels. Contrast agents are substances used in molecular and cellular imaging to amplify the relative differences between targets of interest and background by the absorption of specific electromagnetic wavelengths [26,27]. Contrast agents are often particles that can be designed to take on a variety of forms, which are categorized into organic and inorganic contrast agents. Organic contrast agents, such as indocyanine green (ICG) [28], Prussian blue [29,30], methylene blue [31], and fluorescein sodium [32], are composed of organic compounds, while inorganic contrast agents, such as gold nanoparticles (GNPs) and nanorods are composed of metals or semiconductors. GNPs are useful in enhancing the contrast of OCT and photoacoustic microscopy (PAM) imaging [33-38]. PAM uses light to induce ultrasound waves to be emitted from thermal agents such as gold, which can then be used to reconstruct high-resolution 3D images of vascular and anatomical features [39,40]. The laser fluence utilized for PAM imaging can be used at safe levels for eyes and still create high-resolution and high-contrast images [10,41]. This could possibly allow for early detection of disease onset, possibly resulting in better outcomes. OCT imaging uses the backscattering of light on biological tissue to reconstruct cross-sectional eye images [18].

ICG is soluble in water and has an absorption peak of around 800 nm. Since ICG binds with plasma proteins, it is able to visualize the choroidal vasculature as it does not leak from choroidal blood vessels [42]. To visualize leakage from retinal vasculature, fluorescein can be used. In contrast to ICG, GNPs are inorganic agents that can come in different sizes and shapes, such as rods [36,43-45], stars [35,44,46], clusters [33,38,44], and nanoprisms [44]. The use of GNPs has not yet been approved for human use by the U.S. Food and Drug Administration (FDA). However, studies have shown that ultraminiaturized GNPs are renally excretable and do not cause any discernable damage to organs [33]. Gold has also been shown to have anti-angiogenic properties, as seen by Singh *et al*. [47]. This means GNPs can potentially be used for both imaging and therapy. Using GNPs to enhance contrast can allow for the detection of ocular disease at early stages.

This review will explore advanced contrast agents for eye imaging modalities. While imaging techniques such as PAM, OCT, scanning laser ophthalmoscopy (SLO), and fundus photography all have their upsides and downsides, we will discuss multimodality imaging as a method to overcome shortcomings from any single imaging methodology. We will also discuss the use of organic agents, such as ICG and inorganic contrast agents, such as GNPs, to complement and improve upon these imaging modalities. This includes improving image contrast as well as allowing longitudinal imaging of ocular treatments, such as regenerative cell-based therapies. Finally, we will discuss potential therapeutic uses of contrast agents in addition to their ability to improve image quality.

## Advanced ophthalmic imaging

Medical imaging is highly important for the early detection, diagnosis, and monitoring of diseases and other conditions throughout the body. A wide range of different imaging modalities are currently used in clinical practice to visualize the location and activity of target molecules, cells, and organs. Imaging technology, including positron emission tomography (PET) [48,49], magnetic resonance imaging (MRI) [50,51], single-photon emission computed tomography (SPECT) [52], computed tomography (CT) [53,54], and ultrasound [55], have been applied to numerous areas of medicine, such as neurology, cardiology, and oncology. However, many of these technologies have limited resolution to provide clear, detailed visualization of the retina, choroid, and other structures at the back of the eye. Several current and emerging non-invasive imaging modalities are being applied to optical imaging, and each has strengths and weaknesses that are analyzed in the following subsections.

### Optical coherence tomography (OCT)

Optical coherence tomography (OCT) provides cross-sectional tissue imaging of the retina in ophthalmological applications and works by detection of backscattered photons in the near-infrared region of the electromagnetic spectrum via low-coherence interferometry [18,56,57]. Specifically, OCT produces an image by measuring the echo time delay of the emitted photons, where a longer delay indicates a greater depth of the tissue layer that caused photo reflection. With measured photons being in the near-infrared range, OCT allows for a significant penetration depth of several hundred microns [17,57], in addition to high-resolution imaging (axial resolution ranging from 1 to 15 μm) and real-time visualization [58]. While the possible imaging depth is lower than traditional ultrasound, OCT achieves a 10 to 100 times higher resolution [58]. Spectral-domain OCT (SD-OCT) and swept-source OCT (SS-OCT) advances in OCT technology have led to rapid image acquisition times, with scan rates greater than 100 kHz in commercially available OCT systems, minimizing motion artifacts [57,59,60].

High sensitivity is also a key feature of OCT imaging, where imaging sensitivity is characterized by the greatest attenuation of the signal picked up by the sensor that can still be differentiated from background noise. Common SD-OCT and SS-OCT systems are able to image at sensitivity levels greater than 100 dB, allowing for the detection of structures with very low reflectivity [57]. However, OCT imaging sensitivity is inversely proportional to scan rate [61] and also rolls off with deeper scans [57,62]. To reduce these tradeoffs, molecular contrast agents are used in research contexts to increase the strength of signal detection. Additionally, newer OCT technologies, such as nanosensitive OCT (nsOCT), significantly improve imaging sensitivity and resolution to enhance diagnostic capabilities, although preliminary studies have only been used to image anterior eye structures as opposed to deeper structures such as the retina [63,64]. Due to fast scan rates and high sensitivity, OCT has low exposure time and uses light output below established laser safety guidelines for eyes [65,66].

OCT remains one of the most commonly used imaging modalities in research and clinical settings due to its effectiveness and low cost relative to other imaging technologies [18]. Yet, the upfront purchase price of OCT imaging systems remains high and serves as a significant investment for many offices and laboratories. In light of increasing accessibility to OCT technology, a new, low-cost, hand-held OCT system has been in development at Duke University and promises to significantly improve affordability while maintaining current research and clinical standards [67,68].

OCT was originally developed for ophthalmology as a way to visualize structures beyond the exposed, superficial surfaces of the eye *in vivo*. Since then, its use has expanded to a variety of fields, including dermatology [68-70], cardiology [71-73], neurology [74], oncology [75,76], and numerous other fields of medicine. In ophthalmology, the most common clinical applications of OCT are the diagnosis and monitoring of chronic eye conditions, such as age-related macular degeneration (AMD), glaucoma, diabetic retinopathy, as well as acute conditions, such as retinal detachment [77-79]. As OCT has evolved over the past few decades, it has significantly increased in both usage and applicability to various areas of ophthalmic imaging.

### Photoacoustic microscopy

Photoacoustic microscopy (PAM) serves as a non-invasive, non-ionizing imaging modality that has biological applications ranging from organ- to cell-level visualization. Based on the photoacoustic effect, PAM utilizes a short-pulsed laser to illuminate the targeted tissue, generating rapid temperature increases, which results in local thermal expansion [39,80]. Energy from this thermal expansion is converted into ultrasound waves, which can be detected by an ultrasound transducer and converted into electrical signals to form a digital image. As a result, PAM is capable of obtaining both structural and functional information during imaging without the need for exogenous contrast agents [81]. One of the greatest strengths of photoacoustic imaging is its deep imaging capabilities, with tissue penetration depth of up to 10 cm [82]. The tunable laser systems associated with PAM imaging allows for control over the penetration depth from macroscopic to microscopic resolutions [83]. These depths are possible due to the generation of ultrasound waves, which experience less scattering compared to traditional light-based imaging systems. Furthermore, PAM can achieve an aerial axial resolution as small as 2.5 μm and lateral resolution down to 0.4-0.7 μm, allowing for diagnostic and monitoring capabilities of very small structures and cells within the eye [84]. However, resolution decreases with increasing imaging depth.

Imaging speed is one of the technical limitations of PAM, as laser repetition rate and physical scanning speed are difficult to increase [81,85]. Average imaging speed is also dependent upon the combination of components used for each PAM imaging system, which tend to be highly modular in nature. Ongoing research to develop more efficient scanners will bring reductions in image acquisition times. High sensitivity is characteristic of PAM, as PAM offers 100 % relative optical absorption, which is greater than that of OCT by two orders of magnitude [83,86]. The high sensitivity detection of endogenous contrast agents such as hemoglobin in blood allows for clear, high-contrast imaging, although the use of exogenous contrast agents can further enhance image quality [37,87].

PAM is a relatively new imaging technology. There are a limited number of commercially available PAM imaging systems, and no clinical PAM systems exist for eye applications at this time. PAM serves as a safe imaging modality because it does not produce ionizing radiation, and the energy levels emitted from the laser diode can be significantly below American National Standards Institute (ANSI) laser safety limits. Recent studies by Li *et al.* and Zhang *et al.* performed a comprehensive safety evaluation of a PAM imaging system used to visualize retinal blood vessels and showed that pulsed-laser energy levels at 1.6 nJ (1 % of the ANSI safety limit) were able to generate defined images of the retinal vasculature without damage as per histological analysis [88,89], demonstrating that ultralow energy PAM systems can likely be applied safely in the future in clinical eye settings. In addition to uses in ophthalmology, PAM can be used for a variety of imaging applications, including oncology [90,91], neurology [92,93], dermatology [94], and gastroenterology [95]. While still a new and ongoing development, PAM imaging shows promise as an ophthalmic modality capable of safely producing images of high resolution and penetrance for the diagnosis and tracking of a variety of eye conditions. Multiple animal studies have shown excellent PAM imaging outcomes [88,96-98]. For example, in a study evaluating the progression of choroidal vascular occlusion (CVO), PAM imaging was able to successfully visualize CVO at high resolution over the course of 28 days [82].

### Combined optical coherence tomography and photoacoustic microscopy

**Figure 1.**
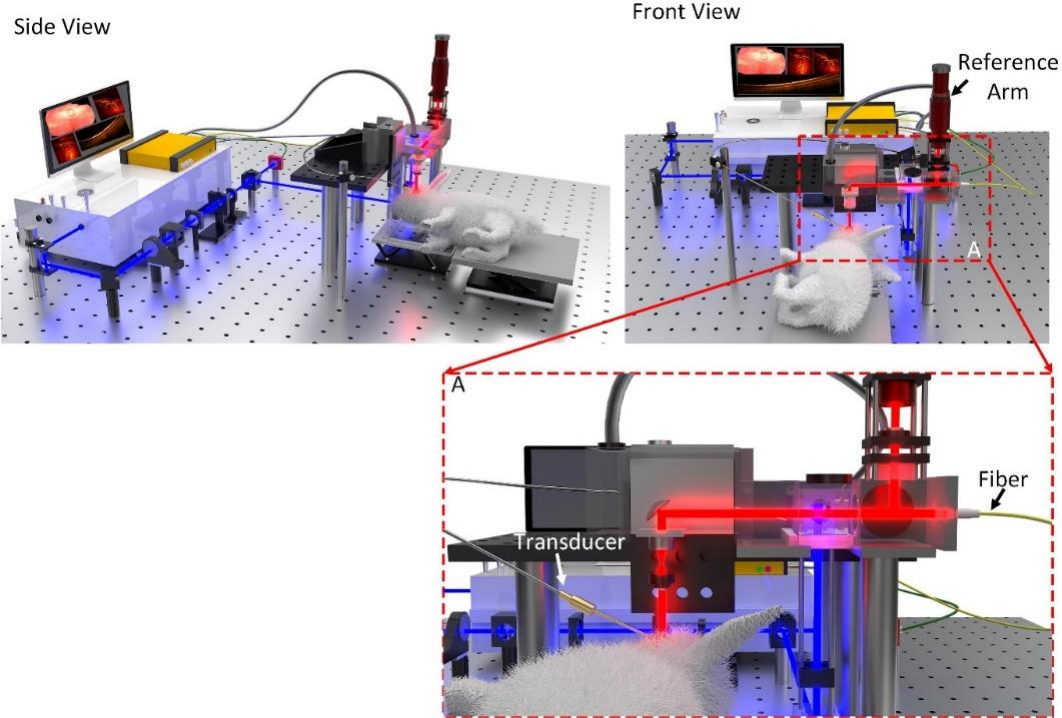
Schematic of multimodal PAM and OCT imaging system. Based on the reference [99].

With efficient and effective diagnostic imaging being crucial to detecting and treating eye diseases, a number of studies have explored the potential of combining the high resolution and fast imaging times of optical coherence tomography (OCT) with the deep penetration depths and high sensitivity of photoacoustic microscopy (PAM) for research applications. These studies commonly combine commercially available OCT systems with components used to create a PAM imaging system, effectively developing a multimodal OCT and PAM system. The integration of OCT and PAM imaging results in overall enhanced imaging characteristics, such as depth, sensitivity, and resolution (Figure 1).

Bondu *et al.* [100] developed a multimodal OCT and PAM system that utilizes a single supercontinuum source paired with a dual-band filter capable of emitting light of wavelengths from 500 to 840 nm or in the infrared light spectrum from 850 to 2300 nm. Through experimentation, it was found that this system was able to achieve a 12.3 μm axial resolution with the OCT channel of the system and a 34 μm axial resolution with the PAM channel, while the OCT could attain a 10.1 μm lateral resolution and an 8.76 μm lateral resolution for PAM [100]. For the OCT channel, the maximum imaging depth was measured as 4 mm in air, with a 3 dB signal attenuation present at an axial distance of 1.3 mm in air [100]. The OCT channel had a signal-to-noise ratio of approximately 35 dB, corresponding to a system sensitivity of 65 dB, and the PAM channel had a signal-to-noise ratio of 20-30 dB. The imaging speed depended on a pulse repetition frequency of 20 kHz of the supercontinuum source. Bondu *et al.* [100] tested the combined OCT and PAM system *in vitro* for synthetic phantoms, each consisting of 3 different dyes, and a mouse ear. The experiments found that for the synthetic phantoms, PAM was more effective than OCT in differentiating between the different dyes in the same sample, while OCT was strong in determining the structural features of the mouse ear, stressing the importance of the multimodal capabilities of the system.

Similarly, Tian *et al.* [16] created a dual-modality OCT and PAM imaging system from commercially available systems and components, which uses a laser that is tunable from 405 to 2600 nm for PAM and two superluminescent diodes at 846 nm and 932 nm. For the system, the quantified aerial axial resolutions were 4.0 μm for OCT and 37.0 μm for PAM, while OCT was able to achieve a 3.8 μm lateral resolution, and PAM was able to achieve a 4.1 μm lateral resolution. This dual-modality system was tested *in vivo* in New Zealand White rabbits and could successfully distinguish single retinal and choroidal vessels with high spatial resolution. The speed of image acquisition of both modalities amounted to approximately 65 seconds, which was limited by the 1 kHz laser repetition rate of the optical parametric oscillator (OPO) laser source for PAM. In addition to the successful visualization of retinal structures and function with the dual-modality system in rabbit eyes, which are similar in size as compared to human eyes, it was found that laser energy used for PAM imaging was approximately 80 nJ, which is less than half of the established ANSI safety limit, making it possibly translatable to clinical use. A more recent study conducted by Nguyen *et al.* [101] longitudinally monitored induced choroidal neovascularization (CNV) in rabbits of different age groups before and after intravitreal bevacizumab treatment using OCT and PAM dual-modality imaging. Results from this study showed that OCT imaging could clearly distinguish the different layers of the choroid and retina in addition to the formation of CNV prior to bevacizumab treatment. Following bevacizumab treatment, OCT was able to provide quantifiable CNV thickness measurements, while PAM imaging combined with chain-like cluster gold nanoparticles (CGNPs) performed after treatment achieved high contrast and spatial resolution for visualization of retinal morphology and vasculature of the choroid. Furthermore, signals detected in the PAM images allowed Nguyen *et al.* to compare signal strength detected from imaging CNV in rabbits of different age groups, demonstrating the direct applicability of combined modality imaging systems. Just as standalone OCT and PAM imaging significantly benefit from the use of organic and inorganic contrast agents, contrast agents can be used to support imaging with combined OCT and PAM dual-modality systems, as already shown in multiple studies [38,102].

A number of other studies have demonstrated the safe and effective imaging capabilities of combined OCT and PAM imaging systems to visualize the eye *in vivo* [8,102,103] as well as conditions in the eye, such as retinal neovascularization [96] and choroidal neovascularization [96,102]. Such multimodal imaging systems also have potential applications in endoscopic imaging, vascular imaging [104,105], and even cancer theranostics [106] that can benefit from the high contrast and deep penetration of these combined systems.

### Combined optical coherence tomography, photoacoustic microscopy, and fluorescence microscopy

Studies have also reported combining OCT, PAM, and fluorescence microscopy (FM) into a single multimodality imaging system capable of providing a more comprehensive visualization that builds on the strengths of each individual imaging modality. Many specifics of OCT and PAM have been presented in previous studies, as described in the subsections above. Similar to dual-modality OCT and PAM systems also being developed, studies on how OCT, PAM, and FM modalities can work together in a single system are relatively few. FM imaging serves as one of the most common modalities used in research and clinical practice to visualize the dynamic aspects of molecular processes *in vivo* [107]. Briefly, FM works by using a camera, microscope, or other light-detecting technology, such as photodiodes, in combination with different excitation and emission filters to capture light that is emitted from excitation of the target following interaction with shorter wavelength incident light. FM imaging can be conducted using two-photon microscopy, which affords high-resolution visualization at the cellular and subcellular levels by using infrared wavelengths that penetrate greater depths into tissue [108-110], but light scattering serves as a significant limitation to the possible imaging depth. Additionally, a signal from induced fluorescence photons decreases with depth due to the combination of fewer fluorescence photons being induced and scattered [108]. Therefore, although FM imaging is popular because of its significant imaging selectivity [108], adaptability, and many applications within ophthalmology and other fields [111], it suffers from the lack of scalability of depth that can be achieved with PAM [112]. As a result, the combination of OCT, PAM, and FM can provide comprehensive visualization capabilities that use the strengths of each individual modality to address the limitations of the other two.

A small number of studies have previously described the details of OCT, PAM, and FM multimodality imaging systems [13,113-116]. Each of these studies has developed a combined modality system that was tested *in vivo*. Dadkhah and Jiao [115] combined PAM, OCT, and confocal fluorescence microscopy (CFM), where the PAM and CFM channels shared the same laser source of a wavelength of 532 nm, maximum pulse energy of 20 μJ, pulse duration of 2 ns, and a maximum pulse repetition rate of 30 kHz. The fluorescence microscopy was based on a confocal system and was limited to a detection range of 550 to 750 nm. This combined system was tested by imaging the ear of a Swiss Webster mouse *in vivo*, yielding a field of view (FOV) of 10×10 and an image acquisition time of 5 minutes. The OCT B-scan images showed depth-resolved tissue structures and sebaceous glands were able to be distinguished due to the shadowing effect of OCT. PAM demonstrated clear visualization of blood vessels of the ear due to contrast produced from optical absorption of hemoglobin, and FM images showed the distribution of sebaceous glands of the ear. Images from the three modalities were transposed on one another to create a single composite image that provided easy visualization of multiple features at once.

Zhang *et al.* [13] produced a multimodality OCT, PAM, and FM imaging system that was applied to visualize retinal neovascularization (RNV) in New Zealand White rabbits and Dutch-Belted rabbits (pigmented) before and a series of days after intravitreal vascular endothelial growth factor (VEGF) injection. The aerial axial resolution of the OCT was 4.0 μm and a lateral resolution was 3.8 μm, while the axial resolution of the PAM channel was 37.0 μm and a lateral resolution was 4.1 μm. This study used a laser wavelength of 905 nm and laser energy of 1.25 mW for OCT, a laser wavelength of 532 nm and laser energy of 80 nJ per pulse for PAM, and a laser wavelength of 480 nm and laser energy of 2 nJ per pulse for FM. Except for OCT, these parameters were well below the ANSI safety limit, with the laser energy of PAM being 60 % below the limit. With alternative, clinically available OCT systems, the light intensity used for OCT imaging can be reduced to meet established safety standards. Before VEGF injection to induce RNV, PAM imaging in this study clearly showed the retinal vasculature, where the main vessels and vascular branches can be easily distinguished. Following induced RNV, PAM was able to provide visualization of individual retinal vessels as well as a new network of small, irregular neovascularization. Prior to RNV, B-scan OCT images showed clearly defined retinal layers in addition to main vessels and vascular branches that could be differentiated from one another. A thick network of blood vessels located on the inner retinal surface could be observed and distinguished from normal vasculature based on differences in shadowing as RNV formed after VEGF injection, although this distinction of individual vessels was more difficult than with PAM. For FM prior to VEGF injection, retinal vessels could be differentiated from the background, although the contrast was much greater in the pigmented rabbits than in the albino rabbits due to less choroidal hyperfluorescence. FM images taken after VEGF injection showed significant progressive hyperfluorescence with blurred margins consistent with the observed leakage of fluorescein dye in angiogenesis, which was once again more easily detected in pigmented rabbits. The image results from this study demonstrate how OCT, PAM, and FM can be linked together to provide a more complete visualization of retinal features and how these features change over time in response to complications of the eye similar to those in humans.

Similar to single- and dual-modality imaging systems, contrast agents can be used to promote high contrast and spatial resolution further in these more extensive multimodal imaging systems that combine OCT, PAM, and FM.

## Contrast agents for ocular imaging

### Organic contrast agents

**Figure 2.**
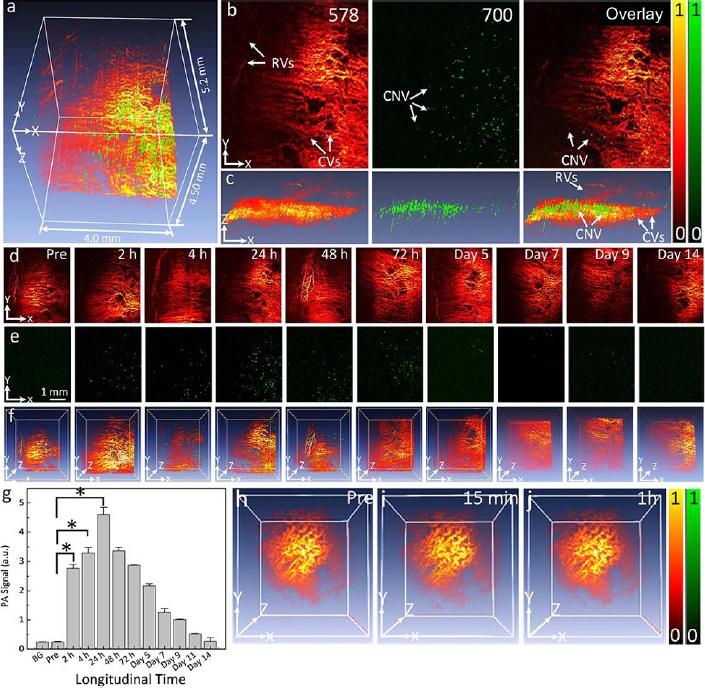
PAM imaging of choroidal neovascularization in rabbit model: **(a)** Merged 3D visualization PAM image of CNV acquired at two different excitation wavelengths of 578 (pseudo-red) and 700 (pseudo-green) nm (post 24 h). **(b)** Horizontal (x-y) PAM image (post 24 h). **(c)** Vertical (y–z) PAM image (post 24 h). **(d-f)** Longitudinal PAM images visualizing CNV obtained at the excitation wavelengths of 578 nm (d) and 700 nm (e) pre-injection of ICG-RGD, and post-injection of ICG-RGD (0.4 mL, 2.5 mg/mL) at different time points. (f) Overlay 3D PAM images. **(g)** Graph of the measured PAM signal in the CNV. **(h-j)** In vivo overlay 3D PAM images of CNV acquired at 578 and 700 nm pre- and post-administration of 0.4 mL ICG without conjugation with RGD at concentration of 2.5 mg/mL at 15 min, and 1 h. BG: background, PA: photoacoustic signal, and pre: PA signal pre-injection. Based on the reference [102].

Optical imaging methods, such as photoacoustic microscopy and optical coherence tomography, are often enhanced using contrast agents. Several organic contrast agents have been widely used for biomedical applications such as Prussian blue, near-infrared (NIR) dyes, carbon nanotubes, indocyanine green, lipid nanoparticles, and nanodroplets. These organic contrast agents can have varying absorption spectra by modifying the size and shape of their molecular structure. Weber *et al.* [26] have shown the benefits of using small-molecule NIR dyes to image cells using PAM. The NIR dyes are small, organic molecules with a variety of electron donor groups attached. Absorption spectra in NIR dyes can be adjusted by modifying, adding, or removing donor groups from the molecule. Carbon nanotubes also have absorption spectra in the NIR range and have significant potential in PAM imaging due to their broad range of possible covalent modifications. Kim *et al.* [30] have studied Prussian blue nanoparticles (PBNPs) in tracking stem cells. The absorption spectra for PBNPs lie in the NIR range, with a maximum absorption peak at 715 nm. The labeled cells remained intact and viable. While many biomedical contrast agents are available, not all are approved for human ocular use. Two organic contrast agents that are applicable to ocular imaging are indocyanine green and nanodroplets. Nguyen *et al.* [102] investigated applications of indocyanine green (ICG) in visualizing choroidal neovascularization (CNV). ICG can be used as a contrast agent for photoacoustic microscopy (PAM). CNV is characterized by the formation and growth of abnormal blood vessels in the eye. Detection of CNV is important because it is the primary cause of vision loss in age-related macular degeneration (AMD). ICG can be conjugated with RGD peptide to form ICG-RGD and allow binding of integrin expressed in CNV for CNV detection. ICG-RGD is administered intravenously and is capable of targeting CNV for up to 5 days post-injection (Figure 2).

PAM imaging shows that the contrast increases to a maximum at 24 hours post-injection. Injection of ICG-RGD in rabbits increases PAM contrast levels by a factor of 15.7 when compared with injection of RGD targeting peptides alone.

Nguyen *et al.* [34] have shown that ICG can also be used in stem cell labeling for subretinally injected progenitor human retinal pigment epithelium cells (ARPE-19). Chain-like gold nanoparticles (CGNP) are conjugated with RGD peptides on their surfaces (CGNP clusters-RGD) along with ICG to create ICG-CGNP clusters-RGD. These particles have a diameter of 56.0 nm and are capable of labeling ARPE-19 cells for regenerative cell-based therapies. Stem cells are emerging as a promising treatment for currently uncurable diseases. The ability to non-invasively track the movement of stem cells is vital to assessing their efficacy.

Zhang *et al.* [117] have researched the imaging applications of nanodroplets, which are microscopic packages in which contrast agents can be carried within tissue. Coating the shell of a nanodroplet with lipid nanoparticles allows for increased biocompatibility. Nanodroplets are a favorable contrast agent due to their high biocompatibility and nontoxicity. They produce a strong photoacoustic signal when encapsulating a photo-absorber molecule. Another advantage comes from their compatibility with a wide range of contrast agents, permitting a great amount of modification. The disadvantages of using nanodroplets are their lack of stability and high rate of photobleaching.

### Nanoparticles in ophthalmology

**Figure 3.**
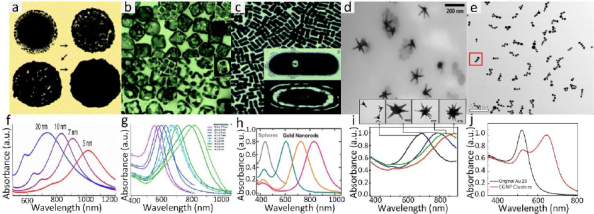
Morphology and optical properties of various GNPs. **(a)** Gold nanoshells (GNS). **(b)** Gold nanocages (GNC). **(c)** Gold nanorods (GNR). **(d)** Gold nanostars (GNST) **(e)** Gold nanochain-like clusters (CGNP). Corresponding UV-Vis absorption spectra of GNS, GNC, GNR, GNST, and CGNP of different aspect ratios, respectively. Adapted with permission from ref.[38,131-133]

Gold nanoparticles (GNPs) currently stand as the most extensive category of contrast agents used in molecular photoacoustic imaging (PAI) and OCT. GNPs possess several characteristics that render them valuable for various biomedical applications. They exhibit remarkable versatility in terms of chemical, physical, and biochemical properties, along with significantly enhanced molar extinction coefficients when compared to small-molecule dyes. In addition, GNPs also exhibit enhanced scattering efficiency as well as the rapid conversion of absorbed light into heat under laser irradiation [26]. GNPs can be found in various shapes (including nanosphere [37,118], nanorods [119,120], nanoplates [121], nanowires, nanodisks [122], nanoshells [123], nanoprisms [124], nanocages [125], nanostars [126], nanovesicles [127,128], and nano bipyramids [129]) and sizes, typically ranging from less than 1,000 nanometers to approximately 10-100 nanometers. GNP’s surface characteristics, reactivity, and optical attributes are adjustable. For example, their extensive surface area allows for the attachment of a considerable number of targeting components per particle, thereby increasing the likelihood of successful target binding. The optical properties (absorption and scattering) can be tuned from visible wavelengths (*i.e.* 520 nm) to the near-infrared (NIR) and second NIR windows (*i.e.* 650 to 1415 nm) through the alteration of both the size and structure of GNPs [45, 130] and the advancement of sophisticated synthesis techniques (Figure 3).

**Figure 4.**
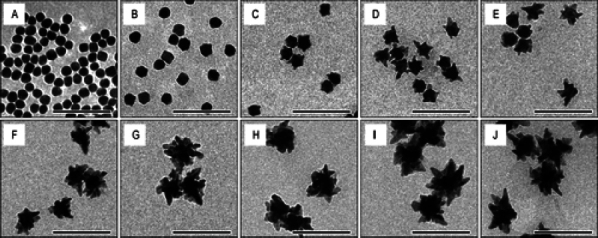
TEM images monitoring the nanostar evolution over time: *t* = **(A)** 0, **(B)** 2, **(C)** 4, **(D)** 6, **(E)** 8, **(F)** 12, **(G)** 16, **(H)** 20, **(I)** 24 and **(J)** 28 min. Adapted with permision from [134] Copyright 2017 American Chemical Society.

The common technique to synthesize GNPs is a chemical method. Chen *et al.* [45] fabricated miniature gold nanorods (GNR) using the seeding solution. By changing the seeding ratio, the length can be adjusted from a large size of 18±4 to 8±2 nm and the width of GNR can be reduced from 120±17 to 49±8 nm while keeping a similar absorption spectrum. Khoury *et al.* [134] have described several methods to control the size and shape of gold nanostars (GNS) from 45 to 116 nm and the longitudinal plasmon peak in the NIR region from around 725 to over 850 nm. By changing the seeding time, the core size, the number, length, and width of branches were increased (Figure 4).

Si *et al.* [129] described gold nanobipyramids with an average length of 137-177 nm and width of 22 to 25 nm as a contrast agent to boost the sensitivity of OCT imaging. Although these methods can synthesize different types of gold nanoparticles with various optical properties, the major challenge is that these GNPs need capping agents like polyethylene glycol (PEG) functionalization [135], stability (*e.g.*, silica encapsulation [136]), active targeting, or CTAB to improve stability [137].

The synthesis process can be complicated. In addition, CTAB is associated with long-term toxicity concerns [137,138]. To address this problem, many alternative methods have been investigated. Cai *et al.* [139] and Katas *et al.* [140] used biocompatible compounds like chitosan as a reducing capping agent for green synthesis of gold nanoparticles, leading to improved stability and avoiding GNP aggregation. Qian *et al.* [141] have introduced a physical method to fabricate gold nanospheres using a femtosecond ablation laser. An advantage of this method is that the fabricated gold nanoparticles have large natural negative charge, and the surface of the GNPs are ultrapure. Thus, these nanoparticles are more biocompatible and exhibit long stability, up to few months, without evidence of aggregation.

A major advantage of GNPs is that they have great localized surface plasmon resonance (LSPR) properties under exposure to light of an appropriate wavelength, which converts a substantial part of the oscillation energy into acoustic waves. The generated acoustic wave can be detected for photoacoustic imaging (PAI). GNPs have been shown as excellent contrast agents for not only PAI but also can be used as drug carriers and have great photothermal effects, which make these nanoparticles promising therapeutic tools for the treatment of various diseases. Recently, the U.S. FDA has approved a gold nanoparticle-coated drug (CNM-Au8, Clene Nanomedicine, Inc.) to treat amyotrophic lateral sclerosis (ALS) through an orally administered route. Moreover, several clinical trials employ gold nanoparticles to address conditions such as atherosclerosis, glioblastoma, and type 1 diabetes [142]. However, due to their larger dimensions, GNPs often accumulate within the reticuloendothelial system for extended periods, leading to reduced biocompatibility. Additionally, achieving consistent, pure, and quantifiable uniform NPs remains a formidable challenge.

### Gold nanoparticle-based OCT contrast agents

**Figure 5.**
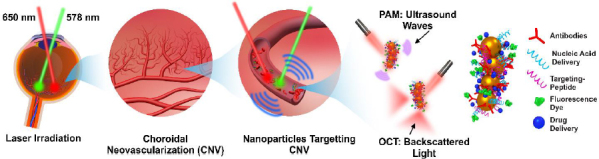
Schematic illustration of gold nanoparticles (GNPs) as multimodality optical coherence tomography (OCT) and photoacoustic microscopy (PAM) image contrast agents for molecular imaging of the eye. Non-targeting (NT-GNPs) and targeting GNPs (T-GNPs) can be administered via intravitreal injection (IVT) or intravenous injection (IV) routes. GNPs' strong plasmonic properties enable them to generate robust back-scattered light and/or acoustic signals when irradiated with an appropriate laser wavelength. These signals are captured by an OCT photodiode to form OCT images or by ultrasound detection to reconstruct photoacoustic (PA) images. Utilizing multiple optical wavelengths within the near-infrared (NIR) window facilitates the detection of GNPs' extravasation at targeted vessels, enabling differentiation of neovascularization.

GNPs show great potential as contrast agents for ophthalmic imaging due to their unique characteristics, ability to reach all parts of the eye, and their eye-friendly clearance behavior. Under light illumination at specific wavelengths, GNPs have the ability to generate strong optical scattering and absorption characteristics due to their highly tunable LSPR properties, which can contribute to enhanced OCT and PAI signal intensity (Figure 5). Numerous types of gold nanoparticles, including gold nanoshells, gold nanostars, gold nanodisks, and gold nanoprisms, have demonstrated a notable capacity to enhance scattering and substantially boost OCT signals.

**Figure 6.**
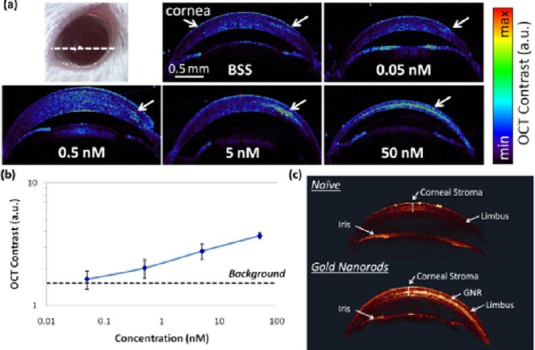
Quantifying GNR detection limit in the mouse cornea. **(a)** Mice corneas were injected with 5 μL of GNR nanoparticles at decreasing concentrations from 50 to 0.05 nM (*N* = 4 total mice), and control mice corneas were injected with balanced saline solution (BSS). Although mice injected with 0.05 nM exhibited OCT contrast similar to control mice, concentrations of 0.5 nM and above produced a distinct detectable OCT signal. The OCT images represent a cross‐sectional slice through the cornea (white dashed line). **(b)** Quantification of the OCT contrast (measured as signal from area of injection divided by corneal signal from an area distant from the injection) was observed to increase with the GNR concentration, with the lowest detectable concentration being 0.5 nM. Below this concentration, the OCT contrast was about equal to the OCT contrast recorded from the control mouse that was injected with BSS (denoted as background in black dashed line). The error bars represent standard deviation of the OCT signal. **(c)** 3D rendering of OCT image. Although naïve mice show minimal signal in the corneal stroma (upper eye), mice injected with 10 μL of GNR at 50 nM show a bright and distinct OCT signal from the corneal stroma (lower eye). The corneal structures (corneal stroma, limbus, and iris) exhibit very consistent OCT signal intensities across both the naïve and injected eyes. Based on the reference [145]. Permissions under Attribution 3.0 International (CC BY 3.0)

A study reported by Prahulkar *et al.* [143] described the use of GNR conjugated with anti-glucose transporter-1 (Glut-1) antibodies for enhanced visualization of ocular surface squamous neoplasia (OSSN) using a commercially available OCT system. Glut-1 is overexpressed in OSSN lesions and selected as a molecular target for GNP functionalization. The acronym OSSN encompasses a range of pathological conditions and lesions, which may involve epithelial dysplasia of the cornea and conjunctiva, carcinoma in situ (CIS), and invasive squamous-cell carcinoma (SCC) [144]. Although gold nanorods have effectively enhanced OCT scattering signals, the choice of molecular markers requires optimization. De la Zerda *et al.* [145] have used GNRs (diameter = 13 nm and length = 45 nm) as contrast agents for OCT in the mouse eye (Figure 6). The GNRs at different concentrations were injected into the anterior chamber and cornea of mouse eyes. The OCT signal intensity was linearly increased as a function of concentrations (Figure 6b). The OCT signal increased up to 50-fold post-injection of GNRs at a final concentration of 30 nM. The minimum detectable concentration in the anterior chamber of the mouse eye was measured to be 10 pM. The surface modification of GNRs with CD45 antibody was applied for labelling mouse leukocytes [146]. After treatment with functionalized GNRs, the cells were administered into the animal through intravenous injection. Interestingly, single cells could be detected within the retinal microvasculature using the OCT imaging system.

**Figure 7.**
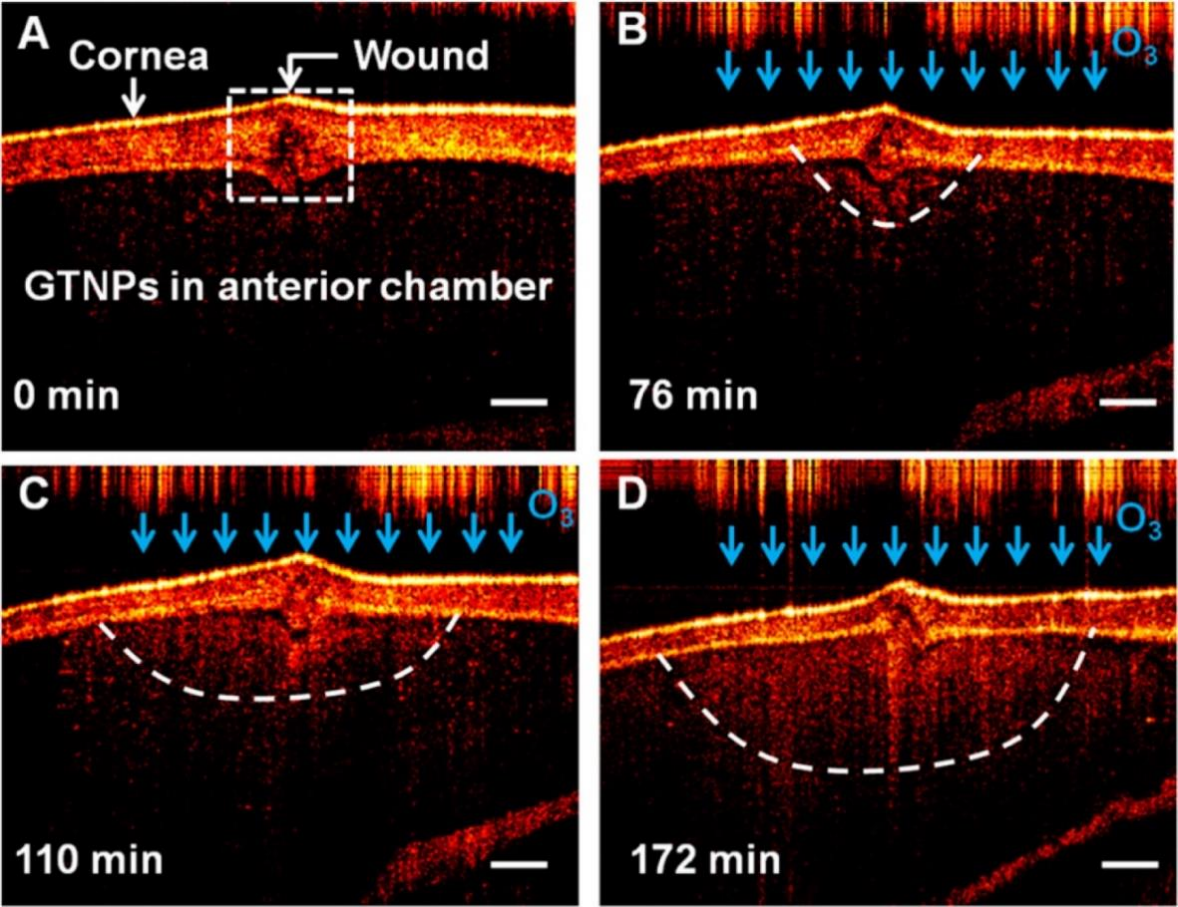
OCT images of an isolated crucian carp eye after the injection of GTNPs in the course of ozone (O_3_) (∼75 ppm) exposure at different durations: **(A)** 0, **(B)** 76, **(C)** 110, and **(D)** 172 min. The cornea of the eye was impaled (shown as the wound marked in panel A). The scale bars represent 300 μm. Adapted with permision from [147] Copyright 2017 American Chemical Society.

There has been a growing interest in researching gold triangular nanoprisms (GTNPs) as contrast agents for OCT, primarily because of their high scattering efficiency. Jiang *et al.* [147] showed that GTNPs with a peak LSPR at 1011, 971, 880, and 810 nm enhanced visualization of damaged tissues caused by ozone (O_3_) on the crucian carp eye. Following the injection of GTNPs at a mass concentration of 0.0228 mg/mL into the anterior chamber of the eye, OCT imaging revealed noticeable contrast enhancement. In the study of the crucian carp eye with a damaged cornea exposed to O_3_, OCT images were captured at various time points. It was observed that the region surrounding the wound exhibited enhanced contrast, attributed to the morphological alteration of the nanoparticles (Figure 7). These findings suggest that gold nanoprisms have the potential to be a promising contrast agent for detecting O_3_ in the eye using OCT imaging.

Lapierre-Landry *et al.* [149] and Gordon *et al.* [149] have demonstrated an advanced photothermal OCT (PT-OCT) with the assistance of GNRs for imaging the retina of laser-induced choroidal neovascularization (CNV) mouse models. Photothermal OCT (PT-OCT) is a method that involves heating a specific tissue region with a laser and light-absorbing contrast agents and then detecting changes in the local refractive index using phase-sensitive OCT. The authors have reported that targeted GNRs with antibodies accumulated at the location of CNV in the eye after injection. Notedly, a significant decrease in PT-OCT signals is observed within the CNV lesion induced by laser after the intravitreal administration of neutralizing monoclonal anti-vascular endothelial growth factor (anti-VEGF) antibodies.

### Gold nanoparticles as contrast agents for photoacoustic molecular imaging

**Figure 8.**
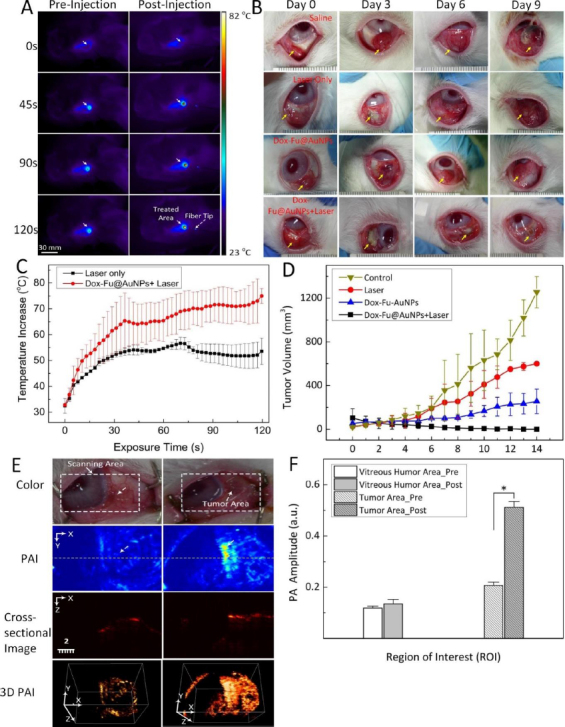
Photoacoustic imaging (PAI) and chemo-photothermal effects of Dox-Fu@AuNPs in rabbit eye tumor model. **(A)** In vivo infrared thermographic maps of eye tumors before and after injection with Dox-Fu@AuNPs followed by laser illumination at different times from 0 s to 120 s. **(B)** Color photographs of the rabbit eye tumors treated with saline as control, laser only, Dox-Fu@AuNPs only, and Dox-Fu@AuNPs with laser irradiation. **(C)** The graph indicates temporal development of temperature from the irradiated area in the tumor during the laser irradiation (laser only vs. Dox-Fu@AuNPs with laser irradiation: N = 20). **(D)** Graph of tumor volume acquired before and after treatment over a period of 14 days. **(E)** Photoacoustic imaging of rabbit eye tumor before and after Dox-Fu@AuNPs injection. **(F)** Quantitative photoacoustic signals obtained from different positions in the rabbit eye tumor before and after injection of Dox-Fu@AuNPs. Based on the reference [150].

Kim *et al.* [150] utilized doxorubicin-conjugated fucoidan-encapsulated GNPs (Dox-Fu@GNPs) for both ocular tumor imaging with PAI and tumor treatment. The PAI contrast within the tumor regions exhibited an increase following intratumor injection of Dox-Fu@GNPs (Figure 8). These nanoparticles were coated with fucoidan and loaded with the chemotherapeutic agent doxorubicin. The combination of chemotherapy and photothermal therapy resulted in a significant reduction in cancer cell viability. The effectiveness of the treatment was dependent on laser fluence and nanoparticle concentration. Using a 532 nm YAG laser irradiation, successful treatment was achieved with irradiance and concentration values of 0.11 W/cm^2^ and 200 μg /mL, respectively. Conversely, no effective treatment was observed at an irradiance of 0.06 W/cm^2^, regardless of Dox-Fu@GNPs concentration. This research demonstrated the utility of GNPs as valuable tools for both treating and imaging intraocular tumors. GNPs enhance PA contrast and assist in delineating the tumor boundaries. Raveendran *et al.* [151] introduced gold nanocages into the irises of *ex vivo* porcine eyes and assessed their PAI contrast properties. This research suggests a possible application of the PAI signal from these nanocages for early detection of uveal melanoma. When used in conjunction with ultrasound imaging, uveal melanoma-targeting gold nanocages have the potential to enhance the precise localization of uveal melanomas within the eye.

Gold nanoparticles can also be used in ophthalmic imaging for visualizing blood vessels. In previous studies, our group has described the feasibility of spherical GNPs coated with polyethylene glycol (PEG) with a diameter of 20 nm as contrast agents to enhance PAM and OCT imaging [37]. After intravenous administration of GNPs at a mass concentration of 5 mg/mL, the PA signal of blood vessels increased by 82 % and the OCT signal increased by 45 % when compared to control. This study demonstrated that GNP-based PAM and OCT imaging allows for improved examination of retinal and choroidal microvasculature.

In another work, our group has developed a novel chain-like cluster of GNPs (CGNPs) conjugated with RGD peptides for multimodal molecular imaging of CNV [38]. A unique property of this CGNP is that we used a femtosecond laser to create the GNPs. Therefore, the synthesized CGNPs have a natural negative charge and the ability to disassemble *in vivo*. In addition, the LSPR absorption peak shifted from 520 nm to 650 nm, allowing for enhanced PAM image contrast at that wavelength due to the low intrinsic PA signal of hemoglobin. When the CGNPs were intravenously injected into the rabbit with the CNV model, the PAM signal increased 17-fold, and the OCT signal increased by 176 %. The CGNPs also enabled the visualization of CNV and distinguished them from the surrounding microvasculature. Similarly, we have compared the benefit of CGNPs with commercially available GNS and GNRs at a similar aspect ratio [35,36]. GNS with an average size of 37 nm (core diameter of 15 nm and tail distance of 22 nm) and LSPR absorption peak of 650 nm and GNRs (width = 34.17±4.85 nm and length = 103.28±12.07 nm) with LSPR absorption peak of 700 nm were administered into the clinical-relevant CNV model in rabbits. Both GNS and GNRs allowed for improved visualization of CNV when compared to strong PAM signal from the background. The PAM signal increased up to 17-fold for GNS, and 27.2-fold for GNRs. The OCT signal intensity increased by 167 % for GNS, and 171.4 % for GNRs.

**Figure 9.**
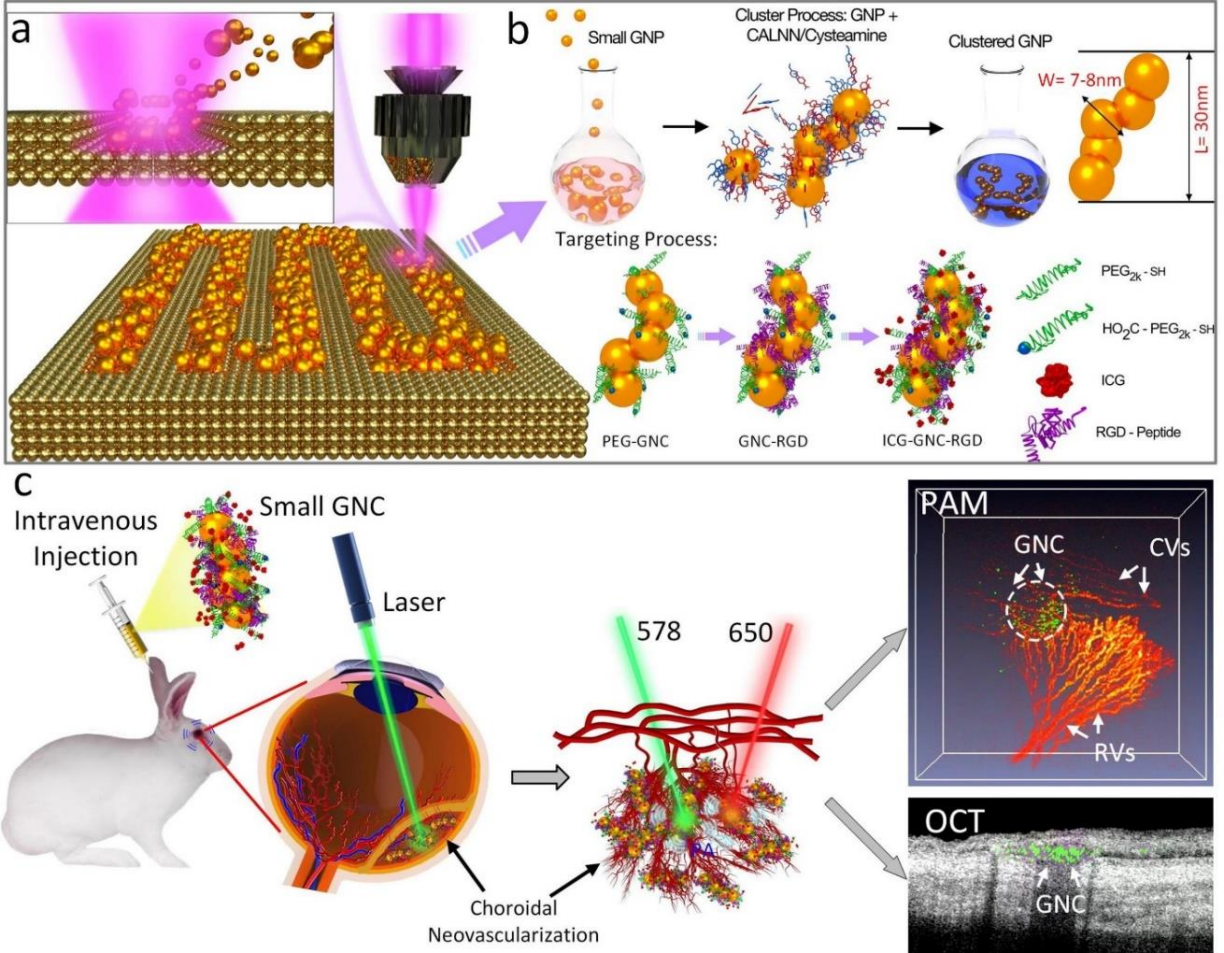
Schematic of the ultraminiature renal clearable GNC encapsulated with RGD ligands (GNC-RGD) and molecular targeting. **(a)** Schematic of physical fabrication of 7-8 nm diameter GNP spheres using pulsed laser ablation (PLA) method. Colloidal 7-8 nm diameter GNP spheres fabricated by the PLA method were self-assembled using cysteamine and pentapeptide Cys–Ala–Leu–Asn–Asn (CALNN) to form a chain-like structure. **(b)** Then, GNCs were encapsulated with RGD ligands (GNC-RGD) and ICG dyes to form ICG-GNC-RGD. **(c)** Illustration of GNC targeting CNV. Under nanosecond pulsed laser, GNC absorb laser energy and induce photoacoustic (PA) signals and OCT signals. Based on the reference [33].

The challenge of these GNPs is that the administered GNPs mainly accumulated in the spleen and liver, leading to long-term safety concerns. De Jong *et al.* [152] have reported that GNPs with a hydrodynamic diameter of less than 10 nm can traverse the blood-brain barrier and accumulate in various organs such as the liver, spleen, testis, lung, blood, and brain. However, GNPs smaller than the filtration cutoff size of the kidney (around 5.5 nm) can be excreted via urine [153]. To utilize this, our group has performed another study by reducing the size of GNPs from 20 to 7 nm and formed an ultraminiature chain structure with a size of 30.0±2.1 nm in length and 7.8±1.1 nm in width (Figure 9) [33].

**Figure 10.**
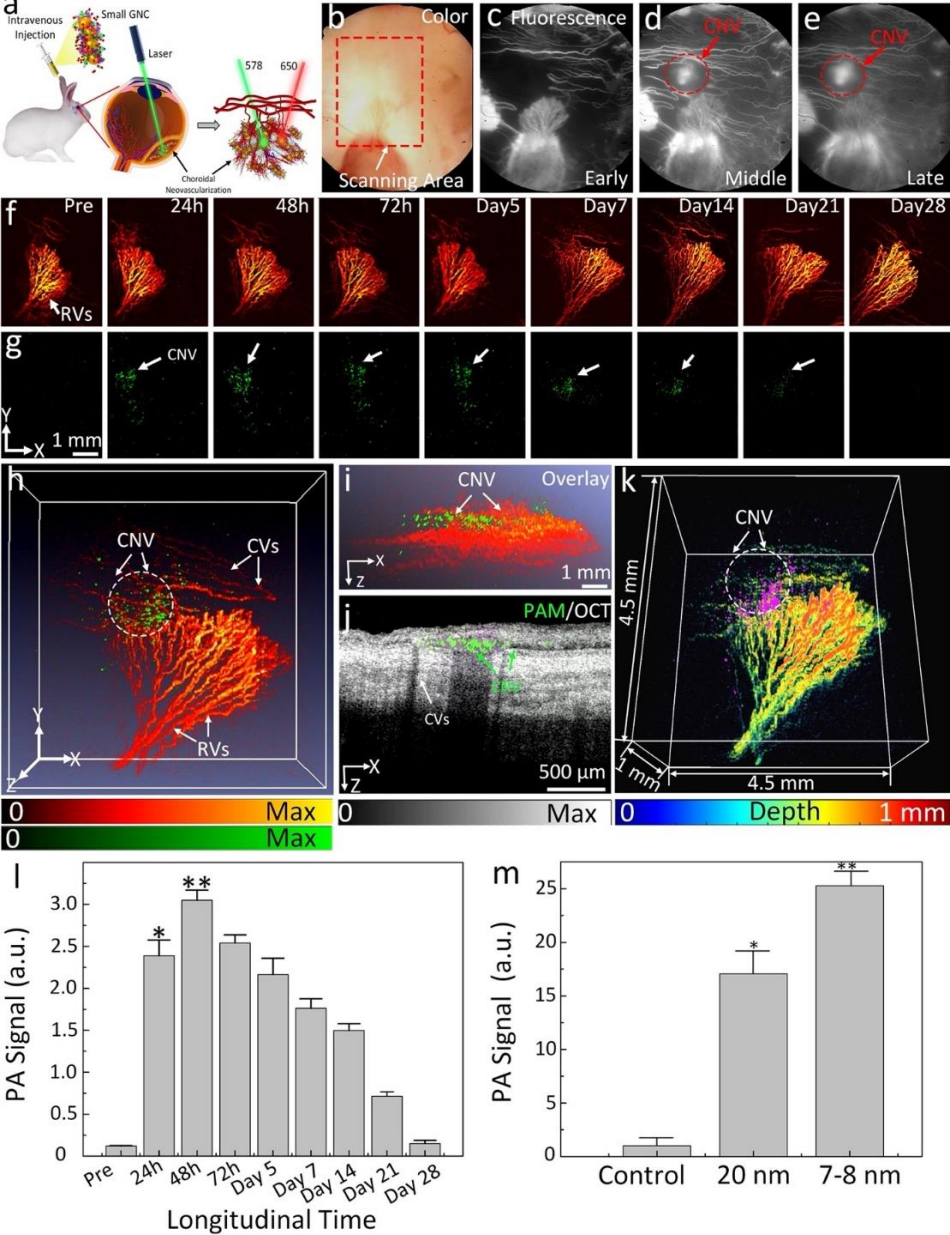
CNV targeting efficiency of ultraminiature GNC in a rabbit RVO model. **(a)** Illustration of GNC targeting CNV. **(b)** Color image obtained at day 28 post laser-induced photocoagulation or RVO model. **(c–e)** Fluorescein angiography (FA) images obtained at different phases: early, middle, and late. Leakage areas at middle and late phase FA demonstrate evidence of the newly developed CNV (red dotted circles). **(f–g)** PAM images obtained along the scanning areas shown in color fundus image (b) at 578 nm (f) to recognize structure of retinal vessels, choroidal vessels, and capillaries and 650 nm (g) to observe choroidal neovascularization (CNV), respectively. **(h)** Selected overlay 3D visualization of CNV obtained at 48 h. The location of CNV (white dotted circle) was clearly observed and matched well with fluorescein angiography (FA) images shown in (c)–(e). **(i)** XZ view of overlay PAM image. **(j)** Co-registered 2D OCT picture and XZ PAM picture obtained at 650 nm. **(k)** Depth-encoded PAM image. **(l)** Graph showing time-dependent PAM signal amplitude measured from the region of interests (ROIs) in (g). **(m)** Comparison of PAM signal amplitude achieved from three different groups: control (non-targeting ultraminiature GNC), targeting ultraminiature GNC, and targeting large GNC. PA: photoacoustic signal, Pre: PA signal pre injection. Based on the reference [33].

**Figure 11.**
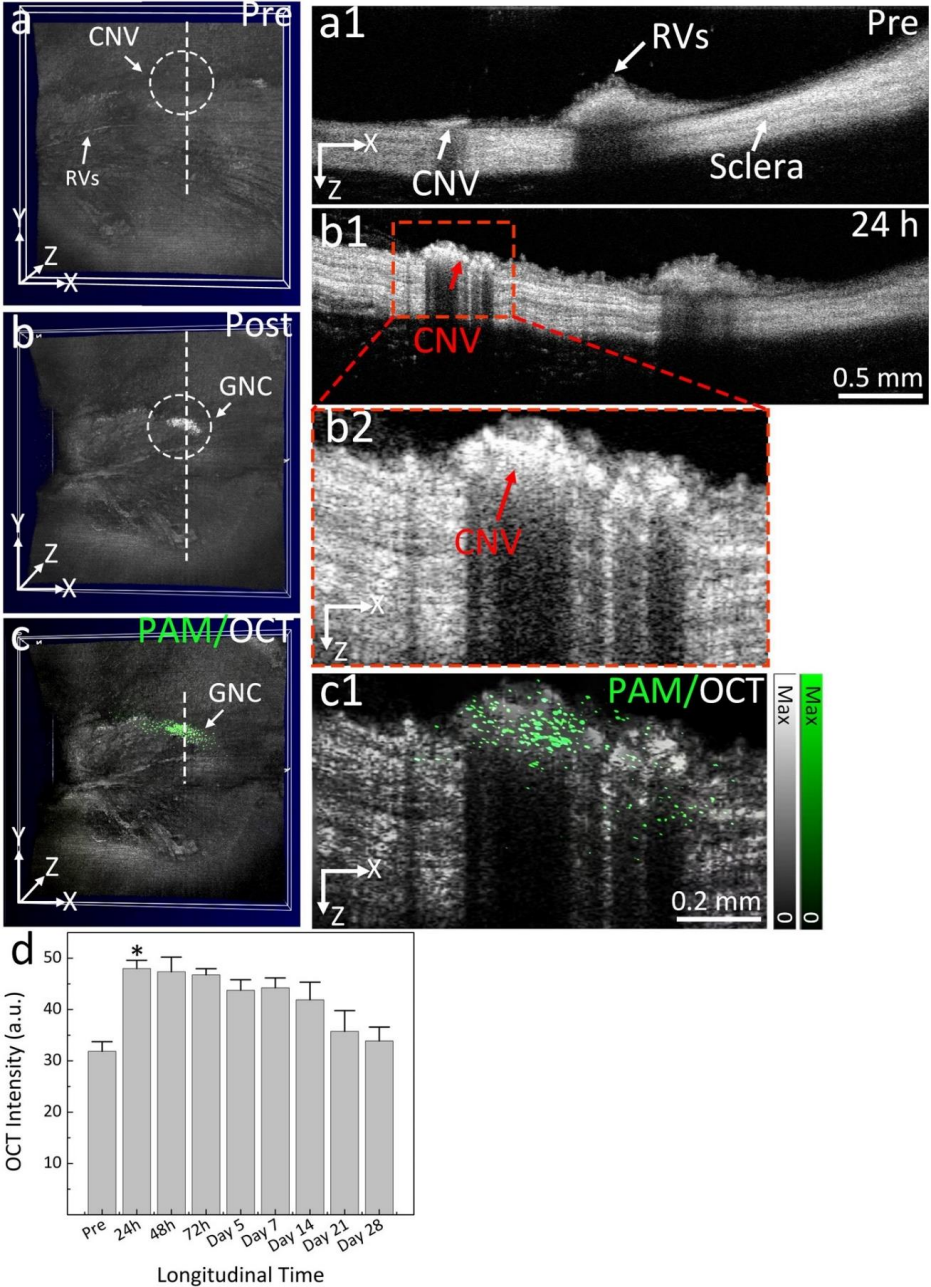
OCT images of CNV post administration of targeting ultraminiature GNC. **(a–b)** 3D volumetric OCT images acquired pre and post administration of ultraminiature GNC, respectively. White dotted circles represent the detected position of newly developed CNV. **(a1)** and **(b1)** 2D OCT images achieved along the scanning dotted line shown in (a) and (b). **(b2)** Magnification OCT image extracted from (b1). **(c)** Co-registered 3D OCT and PAM image achieved at 650 nm. **(c1)** Cross-sectional overlay 2D OCT and PAM. **(d)** Graph of OCT signal intensity measured in the region of interests (ROIs) at various time points post treatment of ultraminiature GNC. Based on the reference [33].

Urine evaluation demonstrated that ultraminiature GNC were able to be excreted from the body via urine while larger GNC, GNPs, and GNR were not excreted in urine. *In vivo* molecular multimodal PAM and OCT imaging demonstrated that ultraminiature GNC prolonged the PAM image contrast for up to 14 days. The image contrast also increased by 25.3-fold for PAM and 150 % for OCT (Figure 10 and Figure 11).

### Gold nanoparticle-enhanced PA imaging tracking of stem cells

Age-related macular degeneration is the leading cause of irreversible blindness in Americans aged 64 and older [3,154,155]. It is estimated that 288 million people will be affected by AMD by 2040 [156]. While treatments like anti-vascular endothelial growth factor (anti-VEGF) and complement inhibition exist, they are unable to reverse geographic atrophy from dry AMD and complement inhibition currently only slows progression. Stem cell therapy (SCT) has the potential to improve vision lost and blinding from several retinal degenerative diseases. Treatment using human induced pluripotent stem cells (hiPSC) differentiated into retinal pigment epithelial (RPE) cells is a promising idea that is being heavily researched. Stem cells can replace cells lost in disease and have the potential to restore vision by replacing dead or degenerated RPE cells in AMD and retinitis pigmentosa (RP) and dying retinal ganglion cells in glaucoma. In 1998, human embryonic stem cells (hESC) and their derivatives were first applied as a new therapeutic approach for several diseases and ushered in a new era of modern medicine [157]. Later, hESC-derived RPE cells were investigated for clinical applications in retinal degenerative diseases, including AMD. The first trial was tested in humans in 2011 and completed in 2017 at multiple world-leading eye centers in the United States, United Kingdom, and South Korea (Clinicaltrial.gov identifiers: UK-SMD: NCT01469832; US-SMD: NCT01345006; US-AMD: NCT01344993). There is no evidence indicating that the transplanted hESC-derived RPE cells caused severe acute or chronic immune inflammation. The immunological rejection was well-managed by injection of immunosuppressants and steroids before and after transplantation. The survival and migration of the transplanted cells were monitored by OCT imaging. There are several routes to deliver stem cells to the retina, including intravitreal injection, subretinal delivery, or suprachoroidal transplantation across the sclera and choroid [158]. However, in order to quantify treatment outcomes, long-term tracking of the migration of stem cells in the eye poses a difficult challenge.

Petrus-Reurer *et al.* [159] utilized human embryonic stem cell-derived retinal pigment epithelial cells (hESC-RPE) in a geographic atrophy rabbit model. Using sodium iodate, the researchers were able to induce RPE degeneration, which was confirmed using OCT imaging. Petrus-Reurer *et al.* observed dose-dependent RPE/Bruch’s/choriocapillaris layer thinning after administration of sodium iodate. After administering hESC-RPE injections to non-treated eyes, Petrus-Reurer *et al.* observed the formation of extensive hESC-RPE monolayers between the photoreceptors and Bruch’s membrane. Over time, the stem cells began to express RPE65 just like native RPE cells. However, this same result was not observed in eyes treated with sodium iodate. The researchers were unable to observe the integration of the hESC-RPE cells into the area of RPE degeneration, most likely due to their chemical-induced RPE damage model. Furthermore, host rejection of hESC-RPE was observed in 3 rabbits. A common hurdle of using cell therapies is the uncontrollable immune reactions that can occur. Immunosuppressants are commonly used to alleviate swelling and infection [160]. Induced human pluripotent stem cells, however, are a potential solution to this issue as they can be collected from the host [161]. Imaging of stem cells was completed in the study with OCT and confocal imaging, but it is limited in terms of long-term tracking of stem cell movement.

Stem cells are difficult to image due to their inherent transparency and lack of signal under most imaging modalities. To visualize stem cells over time after transplantation, Nguyen *et al.* [34] proposed a non-invasive, high-contrast method of tracking stem cells in the eye using chain-like gold nanoparticle clusters (GNCs) along with OCT and photoacoustic (PAM) imaging.

GNCs can be functionalized with RGD peptides and conjugated with ICG to allow for multimodal imaging. Nguyen *et al*. reported a GNC length of 64±21 nm and a width of 20±4 nm. *In vitro* testing of GNCs in ARPE-19 cells shows a minimum detection threshold of 0.01 mg/ml. GNCs are also very stable with minimal photobleaching of only 2 % variance in PAM signal under a 650 nm nanosecond laser at a pulse repetition rate of 1 kHz with a fluence of 0.01 mJ/cm^2^ for 65,000 shots. Since the goal of using GNCs is long-term tracking of stem cells, GNC stability is important. The biocompatibility and cytotoxicity of this contrast agent was also tested. At a high concentration of 100 g/mL and a long incubation time of 48 hours, cell survivability was minimally affected with 80 % viability. Nguyen *et al.* [34] reported an OCT and PAM signal increase of 10-fold using GNC as a contrast agent vs unlabeled ARPE-19 cell. These results are from labeling 10,000 stem cells, almost 1,000 times lower than the typically injected doses of stem cells, demonstrating that the contrast agent is sensitive enough to track stem cell migration in potential regenerative medicine settings.

**Figure 12.**
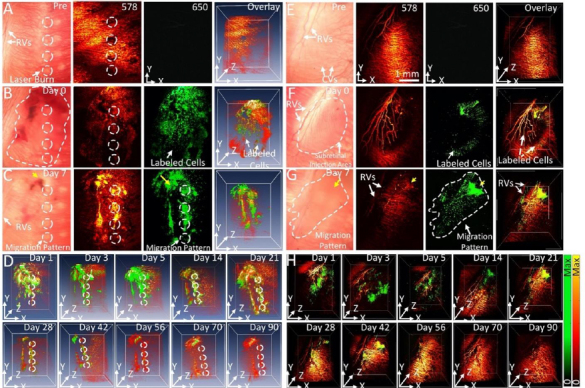
PAM images of labeled ARPE-19 cells post-transplantation into rabbit retina: **(A-D)** PAM images of CGNP clusters-labeled ARPE-19 cells post-transplantation into rabbit eyes with laser-induced RPE injury. (A) Baseline fundus photograph and PAM images acquired at 578 nm and 650 nm before the transplantation of the ARPE-19 cells. White arrows indicate the locations of retinal vessels (RVs) and photocoagulation lesions. (**B-D**) 2D and 3D volumetric PAM images of the ARPE-19 cells acquired at different time points post-transplantation, illustrating cell viability and migration toward photocoagulation lesions. The dotted line in **(B)** indicates the distribution of ARPE-19 cells post-transplantation, while the dotted circles in (**A-C**) show the lesion areas. The pseudo green color in PAM images acquired at 650 nm shows the distribution of the transplanted ARPE-19 cells. **(E-H)** In vivo PAM images of CGNP clusters-labeled ARPE-19 cells post-transplantation into rabbit eyes without laser-induced RPE injury. **(E)** Fundus photograph and PAM images acquired at 578 nm and 650 nm before the transplantation of ARPE-19 cells. **(F-H)** 2D and 3D volumetric PAM images acquired at different time points post-transplantation, illustrating cell viability and migration in a random fashion. The dotted line in **(F)** indicates the distribution of cells immediately post-injection, while the dotted line in **(G)** illustrates cell migration over time. Based on the reference [34].

The feasibility of long-term tracking of stem cells was performed by Nguyen *et al.* [34] in rabbits with laser photocoagulation damage of RPE. The locations of the initial laser burn are marked by the white dotted lines in Figure 12 A-C. PAM imaging of rabbits was performed longitudinally from 1 day to 3 months after injection of labeled stem cells. Cells can be seen migrating towards the areas of laser damage by day 2 after injection. Maximum migration and accumulation of stem cells in the areas of laser damage can be seen by day 7 (Figure 12A-D). In addition to the migration of stem cells, our PAM and OCT imaging allow for visualization of the retinal and choroidal vessels in the eye. At a wavelength of 578 nm, both the stem cells and the ocular vasculature are visible, while at 650 nm, only stem cells are visible (Figure 12A-G). The dual visualization capabilities of Nguyen *et al.*’s imaging system give a better understanding of how stem cells migrate in the eye, as their movement can be referenced to anatomical features when combined with multimodal imaging, such as OCT.

## Conclusion and perspective

Every imaging modality has unique disadvantages and benefits. However, using multiple modalities, one can work around the disadvantages of anyone imaging modality. OCT imaging can provide limited visualization of the choroidal microvasculature and leaking blood vessels, so Nguyen *et al.* [162] furthered imaged with PAM imaging as a solution to these limitations.

Contrast agents can also be compounded to deliver optimized contrast and even therapeutic benefits. Gold nanoparticles can be compounded with ICG to provide even better contrast and light absorption at different wavelengths to better visualize retinal vasculature. Inorganic contrast agents can also be functionalized into therapeutic agents. Gold nanoparticles can be used as thermal agents for photoablation in the eye to target tumors and disrupt membranes for better drug therapy. Peynshaert *et al.* [163] were able to successfully disrupt the inner limiting membrane using ICG as a thermal agent to improve drug therapy to the retinal layers.

Functionalized ICG-gold nanoparticles have also been studied as a contrast agent for longitudinal stem cell imaging in the eye. Since stem cells are transparent and very difficult to image and track, gold nanoparticles have been studied as a potential solution to provide high contrast and long-term imaging of these cells. With increasing regulatory approval of nanoparticles for imaging and therapy, the future of nanoparticles in eye applications is very promising.

The advancement of innovative molecular probes and contrast agents plays a pivotal role in precisely targeting and labeling molecular and cellular structures within the eye. Progress in molecular biology, nanotechnology, and chemistry holds the potential to craft more discerning and responsive probes for ocular imaging. This, in turn, facilitates enhanced detection and characterization of ocular diseases. To fully address the capabilities of ocular molecular and cellular imaging, it is essential to transition these technologies from research settings to standard clinical practice. This entails tackling regulatory challenges, establishing standardized imaging protocols, validating imaging biomarkers, and seamlessly integrating imaging systems into clinical workflows. Overcoming these hurdles and advancing ocular molecular and cellular imaging methods holds the potential to greatly enhance our comprehension, diagnosis, and treatment of ocular diseases. Ultimately, this advancement could result in improved patient outcomes and the creation of personalized treatments in the future.

In conclusion, combining multiple modalities with advanced contrast agents such as gold nanoparticles are the key to providing safer and more extensive imaging data to improve our understanding and treatment of eye diseases. Future directions would include better methods of combining the data gathered from multiple imaging modalities to provide readable and actable data. Using deep learning and computer vision to auto segment OCT data and combining that information with other imaging modalities such as PAM can make large strides in improving outcomes and understanding of disease.
